# Retinoic acid protects from experimental cerebral infarction by upregulating GAP-43 expression

**DOI:** 10.1590/1414-431X20175561

**Published:** 2017-04-03

**Authors:** Y. Li, X. Gao, Q. Wang, Y. Yang, H. Liu, B. Zhang, L. Li

**Affiliations:** 1 Department of Geriatrics, Southern Medical University Zhu Jiang Hospital, Guangzhou, China; 2 Department of Neurology, Southern Medical University Zhu Jiang Hospital, Guangzhou, China; 3 Department of Neurology, First Affiliated Hospital, Sun Yat-Sen University, Guangzhou, China; 4 The Second Affiliated Hospital of Guangzhou Medical University, Guangzhou, China

**Keywords:** Focal cerebral infarction, Growth associate protein 43, Retinoic acid, Nerve regeneration

## Abstract

The aim of this study was to investigate whether exogenous retinoic acid (RA) can upregulate the mRNA and protein expression of growth-associated protein 43 (GAP-43), thereby promoting brain functional recovery in a rat distal middle cerebral artery occlusion (MCAO) model of ischemia. A total of 216 male Sprague Dawley rats weighing 300–320 g were divided into 3 groups: sham-operated group, MCAO+vehicle group and MCAO+RA group. Focal cortical infarction was induced with a distal MCAO model. The expression of GAP-43 mRNA and protein in the ipsilateral perifocal region was assessed using qPCR and immunocytochemistry at 1, 3, 7, 14, 21, and 28 days after distal MCAO. In addition, an intraperitoneal injection of RA was given 12 h before MCAO and continued every day until the animal was sacrificed. Following ischemia, the expression of GAP-43 first increased considerably and then decreased. Administration of RA reduced infarction volume, promoted neurological functional recovery and upregulated expression of GAP-43. Administration of RA can ameliorate neuronal damage and promote nerve regeneration by upregulating the expression of GAP-43 in the perifocal region after distal MCAO.

## Introduction

Due to a lack of effective treatments, most of stroke patients have diverse sequelae. The expression of neural plasticity proteins facilitates recovery from stroke. The growth-associated protein (GAP-43/B-50) plays a critical role in neuronal differentiation, plasticity and regeneration. In developing neurons, GAP-43 is abundantly synthesized, especially in the growth cone, which is accompanied by growth of the axon (1). In mature neurons, GAP-43 is highly expressed in regenerating nerves. When the brain is injured, neurons exhibit sprouting of the lateral branches and responsive regeneration of the axon accompanied by increased expression of GAP-43 (2). Most importantly, expression of GAP-43 is increased in regenerating axons and enhances the sprouting of axons after ischemia (3). Overall, GAP-43 is an important marker for evaluating axon injury and the regenerative response in the mature central nervous system (CNS).

Retinoids (vitamin A) regulate the growth and differentiation of a wide variety of cell types and play a crucial role in the physiology of vision and as morphogenic agents during embryonic development (4,5). Retinoic acid (RA), as an active metabolite of vitamin A, plays an important role in the normal development of CNS, and experimental alteration of retinoic acid signaling leads to several abnormalities including the absence of posterior hindbrain, abnormal dorso-ventral patterning of the spinal cord, and lack of neurite outgrowth from the spinal cord to the periphery (6). RA is not only required for the proper development of the CNS but continues to play an important role in the function of the mature brain as well (7–10). RA is present throughout the brain and retinoid signaling has a physiological role in synaptic plasticity and learning and memory behaviors (8).

A reduced amount of GAP-43 mRNA and protein have been found in the brains of aging or vitamin A-deprived rats. Interestingly, the administration of RA to these rats reversed the reduction in the mRNA and protein levels of GAP-43 and concomitantly alleviated both the relational memory and hippocampal long-term potentiation deficits (11–13). Most importantly, a recent study suggested that RA protected damaged neurons in a model of cerebral ischemia (14). However, the protective mechanisms of RA remain largely unknown. The present study was conducted to investigate whether administration of exogenous RA after distal middle cerebral artery occlusion (MCAO) can regulate the mRNA and protein of levels GAP-43, thereby promoting functional recovery of the brain.

## Material and Methods

### Experimental design

A total of 216 male Sprague Dawley rats weighing 300-320 g from the Experimental Animal Center, Guangdong Academy of Medical Sciences, were used for the study. The rats were housed at a constant temperature (22±1°C) and humidity. Animal care and experimentation conformed to the guidelines of our Institutional Animal Use and Care Committee. These animals were randomly divided into three groups (72 animals each): sham-operated group, MCAO+vehicle group and MCAO+RA group. A focal cortical infarction was induced using a distal MCAO model as reported by Brint et al. (15). Briefly, the MCA was electrocauterized at approximately the level of the rhinal fissure and was occluded immediately distal to the lenticulostriate branches such that the resulting infarction would be confined to the neocortex. In sham-operated animals, the MCA was exposed without occlusion. The rats were allowed to recover from the anesthesia before returning to their cages with free access to food and water. Twelve hours before distal MCAO, the vehicle (polyethylene glycol, NaCl and ethanol (70/20/10)) or retinoic acid (150 µg RA/kg body weight) (12,13) were intraperitoneally injected in the latter two groups, and after the distal MCAO was performed, intraperitoneal injections were administered daily until the animals were sacrificed. RA (all-trans RA; Sigma, USA, No. R2625) was dissolved in a mixture containing polyethylene glycol, NaCl and ethanol (70/20/10) (12,13).

### Neurological and infarction volume examination

**Figure 1:**
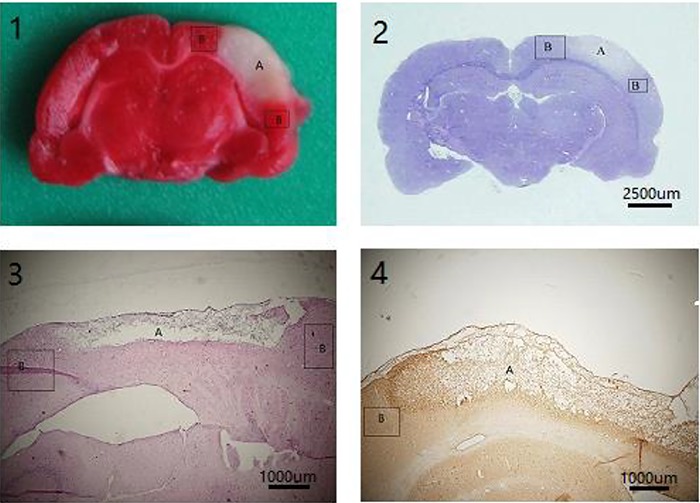
TTC-stained (1), Nissl-stained (2), HE-stained (3), and IHE-stained (4) coronal brain sections of rats showing the infarction area (*A*) and the peripheral regions evaluated (*B*, rectangle indicates the observed location).

Neurological deficit scores were evaluated using the Beam-walking test (16). The rats were deeply anesthetized before they were decapitated, and their brains were cut into coronal slices of 2 mm in thickness. These slices were reacted with a 2% solution of 2,3,5-triphenyltetrazolium chloride (TTC; Sigma Chemical) for 20–30 min to reveal the ischemic infarction (Figure 1). The infarction volume was quantitatively analyzed on hematoxylin and eosin (HE) stained sections obtained from +4.0 to -4.0 mm from the bregma at 2-mm intervals. Data were analyzed with NIH Image 1.31 software for Windows. The following formulas were used: VT = 2.0×Σ[Tn-1+Tn/2]; VS = 2.0×Σ[Sn-1+S n/2], where n≥2, 2.0: 2 mm-interval, VT: whole brain volume, Tn: pixels of the whole brain; VS: infarction volume, Sn: pixel of the infarction region. VS/VT×100% was used to calculate the numerical value.

### Analysis of gene expression using real-time PCR

A total of 108 rats (6 per group) were sacrificed by decapitation at 1, 3, 7, 14, 21, and 28 days after distal MCAO. The brain was rapidly removed and stored at -80°C for subsequent analysis. RNA extraction was performed according to the method of Chomczynski and Sacchi (17) (RNA extraction kit; Invitrogen, USA). The cDNA was synthesized with superscript reverse transcriptase (Tpyobo Co., Ltd., Japan) according to the protocol recommended by the manufacturer. The forward and reverse primer sequences for GAPDH (housekeeping gene) were 5′-GGCAAGTTCAACGGCACAGT-3′ and 5′-CGCCAGTAGACTCCACGACA-3′, respectively. The forward and reverse primer sequences for GAP-43 were 5′-CGACAGGATGAGGGTAAAGAAGA-3′ and 5′-GTGAGCAGGACAGGAGAGGAA-3′, respectively. The primers were designed and synthesized by Takara biotechnology Co., Ltd. (China). The results were normalized by the ratio of the target’s relative concentration to that of GAPDH in the same sample. Real-time PCR cycles were conducted for the amplification of GAP-43 and GAPDH cDNA using a thermal cycler (MJ Research Company, DFC-3200, USA). Real-time PCR was conducted using 2 µL of cDNA in a final reaction volume of 20 µL according to the protocol of the Real Master Mix kit (Tiangen Biotech Co., Ltd., China). The PCR cycling program was set for 1 cycle of pre-denaturation at 95°C for 90 s, then 39 cycles at 95°C for 10 s, 60°C for 30 s, and an 80°C plate reading for 3 s; melting curve data were acquired at every 0.2°C from 55°C to 95°C, and there was a 1-s hold between reads. Because the target and reference (glyceraldehyde 3-phosphate dehydrogenase, GAPDH) gene have different sequences and amplicon lengths, it is probable that they show different PCR efficiencies. Results are reported as the target/reference ratio.

### Analysis of protein expression using immunohistochemistry

A total of 108 rats (6 per group) were sacrificed at 1, 3, 7, 14, 21, and 28 days after distal MCAO. Under anesthesia (*vide supra*), rats were transcardially perfused with 0.9% sodium chloride at 4°C, followed by 4% paraformaldehyde in 0.1 M phosphate-buffer (PB, pH 7.4). The brain was then removed, maintained in the same fixative solution for 4 h at 4°C and immersed in 0.1 MPB containing 20 or 30% sucrose overnight at 4°C. Coronal sections of the brain were obtained at -2.5 to +2.5 mm from the bregma. Twenty-micrometer thick sections were cut on a cryostat (Leica, 2800N, Germany). For immunohistochemistry, free-floating sections were incubated in 0.01 M PBS containing 3% H_2_O_2_ for 30 min. After preincubation for 60 min in 5% normal goat serum, the sections were incubated overnight in primary antibody (GAP-43, 1:400, Sigma). The sections were then rinsed and incubated for 60 min in peroxidase-labeled mouse secondary antibody (ready-to-use, Dako, Japan). The signal was visualized with 3, 3-diaminobenzidine (Liquid DAB, Dako). The negative control sections were incubated in 0.01 M PBS without the primary antibody. The nuclei of the sections were counterstained with hematoxylin. The resulting images from DAB staining were analyzed with a computer-based image analysis system (Image-Pro-Plus, Silver Spring, USA). Every sixth section from -2.5 to +2.5 mm from the bregma was used for quantitative analysis. The integrated optical density (IOD) in the perifocal region was analyzed under 400× magnification. The mean and standard error (SEM) values per field were calculated.

### Statistical analysis

Data are reported as means±SE. Statistical significance was calculated by analysis of variance (ANOVA) followed by Tukey's multiple range *post hoc* test (P<0.05) using SPSS13.0 Statistical Software (USA).

## Results

### Neurological function scores

**Figure 2:**
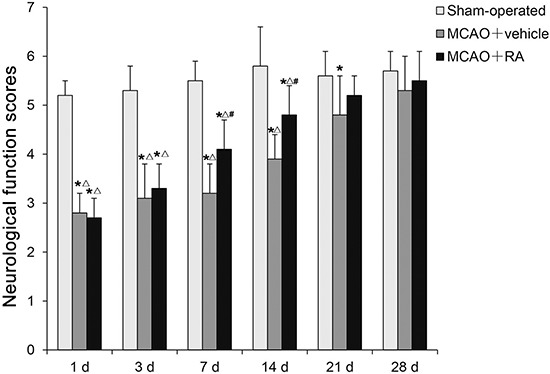
Neurological function scores of rats from the sham-operated group, middle cerebral artery occlusion (MCAO)+vehicle group and MCAO+retinoic acid (RA) group. Data are reported as means±SE. *P<0.05 *vs* sham-operated group; ^#^P<0.05 *vs* MCAO+vehicle group; ^Δ^P<0.05 *vs* 28 days after distal MCAO group (ANOVA followed by Tukey's multiple range *post hoc* test).

Neurological function deficit was observed in ischemic rats but not in the sham-operated rats. The neurological function scores of vehicle-treated ischemic animals at 1, 3, 7, 14, and 21 days were lower than the respective sham-operated animals (P<0.05). The neurological function scores of RA-treated ischemic animals at 1, 3, 7, and 14 days were lower than the respective sham-operated animals (P<0.05). The neurological function scores of RA-treated ischemic animals were higher than those of vehicle-treated animals respectively at 7 and 14 days after MCAO (P<0.05). Vehicle-treated and RA-treated groups showed great improvement in neurological function at 28 days after MCAO, compared with that at 1, 3, 7, and 14 days, which indicated that the neurological function of MCAO rats can be restored naturally. (P>0.05; Figure 2).

### Infarction volume

**Figure 3:**
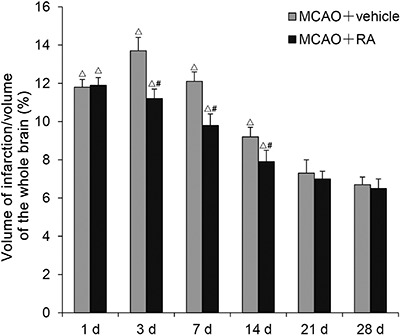
Volume of infarction/volume of whole brain of rats from the middle cerebral artery occlusion (MCAO)+vehicle group and MCAO+retinoic acid (RA) group. Data are reported as means±SE. ^#^P<0.05 *vs* MCAO+vehicle group; ^Δ^P<0.05 *vs* 28 d after distal MCAO group (ANOVA followed by Tukey's multiple range *post hoc* test).

No infarction was observed in sham-operated rats. The infarction volumes of the MCAO+vehicle and MCAO+RA groups at 1, 3, 7, and 14 days were larger than those at 28 days after MCAO (P<0.05). The infarction volumes of the MCAO+RA animals were smaller than the respective MCAO+vehicle animals at 3, 7, and 14 days after MCAO (P<0.05; Figure 3).

### Expression of GAP-43 protein and mRNA in the perifocal region

**Figure 4:**
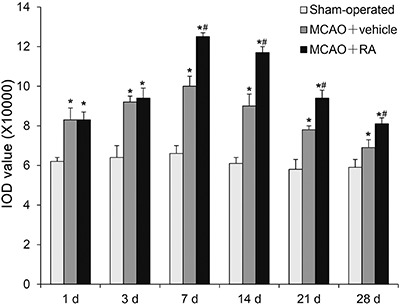
Protein expression of GAP-43 in the perifocal region of rats from the sham-operated group, middle cerebral artery occlusion (MCAO)+vehicle group and MCAO+retinoic acid (RA) group. Data are reported as means±SE. *P<0.05 *vs* sham-operated group; ^#^P<0.05 *vs* MCAO+vehicle group (ANOVA followed by Tukey's multiple range *post hoc* test).

**Figure 5:**
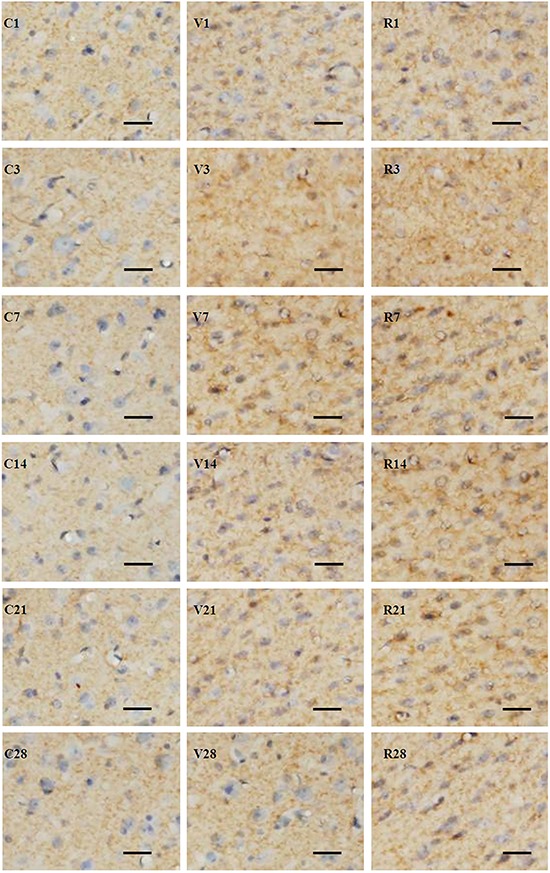
Expression of GAP-43 in the perifocal region of different groups of rats after middle cerebral artery occlusion (MCAO) at different time points (3, 7, 14, 21 and 28 days). The positive signal is distributed in the axon, and is manifested as fine grains in the sham-operated group (C1, 3, 7, 14, 21, and 28), as larger fine grains and floccular grains in the MCAO+vehicle group (V1, 3, 7, 14, 21, and 28), and by increased staining in the MCAO+retinoic acid group (R1, 3, 7, 14, 21, and 28). Frozen sections, immunohistochemistry counterstained with hematoxylin. Scale bars: 50 µm.

**Figure 6:**
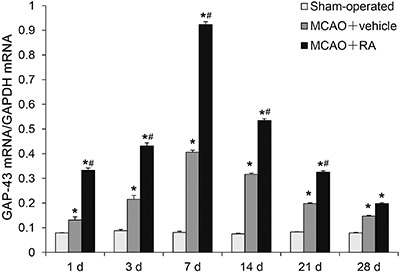
Expression of GAP-43 mRNA in the perifocal region of rats from the sham-operated group, middle cerebral artery occlusion (MCAO)+vehicle group and MCAO+retinoic acid (RA) group. Data are reported as means±SE. *P<0.05 *vs* the sham-operated group; ^#^P<0.05 *vs* the MCAO+vehicle group (ANOVA followed by Tukey's multiple range *post hoc* test).

GAP-43 expression after MCAO in the perifocal region of the MCAO+vehicle group increased to a peak at 7 days after MCAO and thereafter gradually decreased. However, the GAP-43 expression of this group at all time points was higher than that of the sham-operated group (P<0.05). GAP-43 expression in the perifocal region of the MCAO+RA group was much higher than that of the MCAO+vehicle group at 7, 14, 21, and 28 days (P<0.05; Figures 4 and 5). GAP-43 mRNA expression after MCAO in the perifocal region of the MCAO+vehicle group increased to a peak at 7 days after MCAO and thereafter gradually decreased, but the GAP-43 mRNA expression of this group was higher than that of the sham-operated group at all time points (P<0.05). The GAP-43 mRNA expression of the MCAO+RA group was significantly higher than that of the MCAO+vehicle group at 1, 3, 7, 14, and 21 days (P<0.05; Figure 6).

## Discussion

An injury or pathological process that destroys the continuity of axons in the mature CNS has devastating consequences and produces long-lasting functional deficits (18). No valid neuroprotective treatment is available for ischemic infarction. However, stroke patients usually experience some degree of spontaneous improvement of neurological function after a stroke, which may be due to neurite regeneration and synapse rebuilding, as well as the brain edema absorption. Stroke induces a process of axonal sprouting in peri-infarct tissue and it is correlated in location and magnitude with functional recovery after stroke (19–22). A series of plasticity responses after MCAO, including the release of neurotrophic factors, synthesis of neuron-specific proteins and an increase of synaptic excitation, are involved in the process of functional recovery (18,23). However, the spontaneous recovery is eventually impeded by the limitations of neural regeneration and the formation of glial scarring (24). One of the major challenges in this field is to stimulate the re-growth of severed axons and the reconstruction of pathways.

### Effect of RA on the infarction volume and neural function recovery

In the present study, we observed that administration of RA reduced the infarction volume. In addition, although a spontaneous recovery of neurological function deficits occurred after distal MCAO (25), administration of RA significantly increased the speed of the spontaneous neural function recovery. RA reduced the infarction volume possibly by inhibition of inflammation-associated injury (26,27); for example, all-trans-retinoic acid inhibits expression of inducible nitric oxide synthase (iNOS) and several proinflammatory cytokines in activated astrocytes and microglia (28,29). Furthermore, the neuroprotective role of RA may be associated with inhibition of calcium overload during ischemia. Calcium homeostasis is important in maintaining normal cell metabolism. Calcium overload has been found in neurons destined to die during ischemia. Calcium overload triggers the elevation of superoxide radicals and other oxygen radicals. RA signaling can activate protein kinase C (30), which in turn inhibits the intracellular calcium overload after MCAO (31).

### Effect of RA on the expression of GAP-43 mRNA and protein in the perifocal region after distal MCAO

GAP-43 is a neuroaxonal growth protein that is synthesized at high levels during axonal growth in neuronal development and axonal re-growth and regeneration in the peripheral and central nervous system (1,2,32). GAP-43 is mainly distributed in the axonal growth cone and overall expression patterns are under tight developmental control, with cortical GAP-43 expression falling sharply in the adult. When the nervous system is injured, GAP-43 level will be increased (21). Axonal sprouting, an indication of anatomical plasticity, can be identified by the elevated expression of GAP-43. Thus, GAP-43 is considered a main marker of axonal regeneration. The increased expression of GAP-43 may be one of the mechanisms by which functional recovery can be obtained after cerebral ischemia (2). In the present study, the expression changes of GAP-43 mRNA and protein after MCAO are consistent with previous reports (2,33–35).

It has been established that RA is indispensable for the normal development of the CNS of vertebrates (36). More recently, data have shown that the adult brain synthesizes RA (7-9), suggesting that retinoids might play an important role in the adult central nervous system. Moreover, the brain exhibits a selective distribution of cellular retinoid-binding proteins (37) and nuclear receptors (38). RA produces a marked effect by binding to its receptors, which regulate target genes (8). Taken together, our data suggest that administration of RA upregulates the expression of GAP-43 mRNA and protein. Intraperitoneal administration of RA can upregulate its receptors (11,13,39) and these nuclear receptors combine with the specific DNA domains to regulate downstream genes, such as GAP-43 mRNA (8,11,13). Therefore, we speculate that the neuroprotective effects of RA are associated with the upregulation of GAP-43 mRNA and GAP-43 protein expression. These results are in agreement with the findings of Xing et al. (40), in spite of the differences in administration time and dosage. In that study, GAP-43 expression was observed only at 7 days after MCAO.

It should be noted that this study has some limitations, such as RA dosage was not based on pilot study results of dose-effect curves but was based on published studies. The best dosage of RA for the treatment of cerebral ischemia will be further explored.

In conclusion, administration of RA reduced the infarction volume and speed-up neurological functional recovery. The neuroprotective mechanism of RA is attributed, at least in part, to upregulation of GAP-43 mRNA and protein.

## References

[1] Mahalik TJ, Carrier A, Owens GP, Clayton G (1992). The expression of GAP43 mRNA during the late embryonic and early postnatal development of the CNS of the rat: an *in situ* hybridization study. Brain Res Dev Brain Res.

[2] Li S, Carmichael ST (2006). Growth-associated gene and protein expression in the region of axonal sprouting in the aged brain after stroke. Neurobiol Dis.

[3] Nadeau S, Hein P, Fernandes KJ, Peterson AC, Miller FD (2005). A transcriptional role for C/EBP beta in the neuronal response to axonal injury. Mol Cell Neurosci.

[4] Ross SA, McCaffery PJ, Drager UC, De Luca LM (2000). Retinoids in embryonal development. Physiological Reviews.

[5] Mark M, Ghyselinck NB, Chambon P (2009). Function of retinoic acid receptors during embryonic development. Nucl Recept Signal.

[6] Maden M, Hind M (2003). Retinoic acid, a regeneration-inducing molecule. Dev Dyn.

[7] McCaffery P, Zhang J, Crandall JE (2006). Retinoic acid signaling and function in the adult hippocampus. J Neurobiol.

[8] Lane MA, Bailey SJ (2005). Role of retinoid signalling in the adult brain. Prog Neurobiol.

[9] Denisenko-Nehrbass NI, Jarvis E, Scharff C, Nottebohm F, Mello CV (2000). Site-specific retinoic acid production in the brain of adult songbirds. Neuron.

[10] Misner DL, Jacobs S, Shimizu Y, de Urquiza AM, Solomin L, Perlmann T (2001). Vitamin A deprivation results in reversible loss of hippocampal long-term synaptic plasticity. Proc Natl Acad Sci U S A.

[11] Li L, Li Y, Ji X, Zhang B, Wei H, Luo Y (2008). The effects of retinoic acid on the expression of neurogranin after experimental cerebral ischemia. Brain Res.

[12] Feart C, Mingaud F, Enderlin V, Husson M, Alfos S, Higueret P (2005). Differential effect of retinoic acid and triiodothyronine on the age-related hypo-expression of neurogranin in rat. Neurobiol Aging.

[13] Husson M, Enderlin V, Alfos S, Boucheron C, Pallet V, Higueret P (2004). Expression of neurogranin and neuromodulin is affected in the striatum of vitamin A-deprived rats. Brain Res Mol Brain Res.

[14] Harvey BK, Shen H, Chen GJ, Yoshida Y, Wang Y (2004). Midkine and retinoic acid reduce cerebral infarction induced by middle cerebral artery ligation in rats. Neurosci Letters.

[15] Brint SU, Al-Khalidi HR, Vatel B, Hier DB (1996). MCA flow asymmetry is a marker for cerebrovascular disease. Neurol Research.

[16] Feeney DM, Gonzalez A, Law WA (1982). Amphetamine, haloperidol, and experience interact to affect rate of recovery after motor cortex injury. Science.

[17] Chomczynski P, Sacchi N (1987). Single-step method of RNA isolation by acid guanidinium thiocyanate-phenol-chloroform extraction. Anal Biochem.

[18] Stichel CC, Muller HW (1998). Experimental strategies to promote axonal regeneration after traumatic central nervous system injury. Progr Neurobiol.

[19] Dijkhuizen RM, Singhal AB, Mandeville JB, Wu O, Halpern EF, Finklestein SP (2003). Correlation between brain reorganization, ischemic damage, and neurologic status after transient focal cerebral ischemia in rats: a functional magnetic resonance imaging study. J Neurosci.

[B20] Calautti C, Baron JC (2003). Functional neuroimaging studies of motor recovery after stroke in adults: a review. Stroke.

[21] Carmichael ST (2003). Plasticity of cortical projections after stroke. Neuroscientist.

[22] Carmichael ST, Chesselet MF (2002). Synchronous neuronal activity is a signal for axonal sprouting after cortical lesions in the adult. J Neurosci.

[23] Lykissas MG, Batistatou AK, Charalabopoulos KA, Beris AE (2007). The role of neurotrophins in axonal growth, guidance, and regeneration. Current Neurovasc Res.

[24] Panickar KS, Norenberg MD (2005). Astrocytes in cerebral ischemic injury: morphological and general considerations. Glia.

[25] Liu L, Zhu L, Zou Y, Liu W, Zhang X, Wei X (2014). Panax notoginseng saponins promotes stroke recovery by influencing expression of Nogo-A, NgR and p75NGF, *in vitro* and *in vivo*. Biol Pharmac Bulletin.

[26] Malaspina A, Michael-Titus AT (2008). Is the modulation of retinoid and retinoid-associated signaling a future therapeutic strategy in neurological trauma and neurodegeneration?. J Neurochemj.

[27] Mey J (2006). New therapeutic target for CNS injury? The role of retinoic acid signaling after nerve lesions. J Neurobiol.

[28] Xu J, Chavis JA, Racke MK, Drew PD (2006). Peroxisome proliferator-activated receptor-alpha and retinoid X receptor agonists inhibit inflammatory responses of astrocytes. J Neuroimmunol.

[29] Dheen ST, Jun Y, Yan Z, Tay SS, Ling EA (2005). Retinoic acid inhibits expression of TNF-alpha and iNOS in activated rat microglia. Glia.

[30] Evans TR, Kaye SB (1999). Retinoids: present role and future potential. Br J Cancer.

[31] Henriksson M, Stenman E, Vikman P, Edvinsson L (2007). Protein kinase C inhibition attenuates vascular ETB receptor upregulation and decreases brain damage after cerebral ischemia in rat. BMC Neurosci.

[32] Emery DL, Royo NC, Fischer I, Saatman KE, McIntosh TK (2003). Plasticity following injury to the adult central nervous system: is recapitulation of a developmental state worth promoting?. J Neurotrauma.

[33] Miyake K, Yamamoto W, Tadokoro M, Takagi N, Sasakawa K, Nitta A (2002). Alterations in hippocampal GAP-43, BDNF, and L1 following sustained cerebral ischemia. Brain Res.

[B34] Stroemer RP, Kent TA, Hulsebosch CE (1998). Enhanced neocortical neural sprouting, synaptogenesis, and behavioral recovery with D-amphetamine therapy after neocortical infarction in rats. Stroke.

[35] Gregersen R, Christensen T, Lehrmann E, Diemer NH, Finsen B (2001). Focal cerebral ischemia induces increased myelin basic protein and growth-associated protein-43 gene transcription in peri-infarct areas in the rat brain. Exper Brain Res.

[36] Maden M (2001). Role and distribution of retinoic acid during CNS development. Int Rev Cytol.

[37] Zetterstrom RH, Lindqvist E, Mata DUA, Tomac A, Eriksson U, Perlmann T (1999). Role of retinoids in the CNS: differential expression of retinoid binding proteins and receptors and evidence for presence of retinoic acid. Eur J Neurosci.

[38] Moreno S, Farioli-Vecchioli S, Ceru MP (2004). Immunolocalization of peroxisome proliferator-activated receptors and retinoid X receptors in the adult rat CNS. Neuroscience.

[39] Boucheron C, Alfos S, Enderlin V, Husson M, Pallet V, Jaffard R (2006). Age-related effects of ethanol consumption on triiodothyronine and retinoic acid nuclear receptors, neurogranin and neuromodulin expression levels in mouse brain. Neurobiol Aging.

[40] Xing HY, Meng EY, Xia YP, Peng H (2015). Effect of retinoic acid on expression of LINGO-1 and neural regeneration after cerebral ischemia. J Huazhong Univ Sci Technolog Med Sci.

